# Pre-admission interventions to improve outcome after elective surgery—protocol for a systematic review

**DOI:** 10.1186/s13643-016-0266-9

**Published:** 2016-05-23

**Authors:** Rachel Perry, Lauren J. Scott, Alison Richards, Anne M. Haase, Jelena Savović, Andrew R. Ness, Charlotte Atkinson, Jessica Harris, Lucy Culliford, Sanjoy Shah, Maria Pufulete

**Affiliations:** Clinical Trials and Evaluation Unit, School of Clinical Sciences, University of Bristol, Level 7, Bristol Royal Infirmary, Queen’s Building, Bristol, BS2 8HW UK; Biomedical Research Unit in Nutrition Diet and Lifestyle, University of Bristol, Lower Maudlin Street, Bristol, BS1 2LY UK; NIHR CLAHRC West, University Hospitals Bristol NHS Foundation Trust, 9th Floor, Whitefriars, Lewins Mead, Bristol, BS1 2NT UK; Centre for Exercise, Nutrition and Health Sciences, School for Policy Studies, 8 Priory Road, Bristol, BS8 1TZ UK; Preoperative Assessment Clinic, Bristol Royal Infirmary, University Hospitals Bristol NHS Foundation Trust, Bristol, BS2 8HW UK

**Keywords:** Prehabilitation, Elective surgery, Systematic review, Physical activity, Preoperative nutrition, Improving fitness before surgery

## Abstract

**Background:**

Poor physical health and fitness increases the risk of death and complications after major elective surgery. Pre-admission interventions to improve patients’ health and fitness (referred to as prehabilitation) may reduce postoperative complications, decrease the length of hospital stay and facilitate the patient’s recovery. We will conduct a systematic review of RCTs to examine the effectiveness of different types of prehabilitation interventions in improving the surgical outcomes of patients undergoing elective surgery.

**Methods:**

This review will be conducted and reported according to the Cochrane and PRISMA reporting guidelines. MEDLINE, EMBASE, CENTRAL, CINAHL, PsycINFO, ISI Web of Science and clinical trial registers will be searched for any intervention administered before any elective surgery (including physical activity, nutritional, educational, psychological, clinical or multicomponent), which aims to improve postoperative outcomes. Reference lists of included studies will be searched, and grey literature including conference proceedings, theses, dissertations and preoperative assessment protocols will be examined. Study quality will be assessed using Cochrane’s risk of bias tool, and meta-analyses for trials that use similar interventions and report similar outcomes will be undertaken where possible.

**Discussion:**

This systematic review will determine whether different types of interventions administered before elective surgery are effective in improving postoperative outcomes. It will also determine which components or combinations of components would form the most effective prehabilitation intervention.

**Systematic review registration:**

PROSPERO CRD42015019191

## Background

Each year in England, there are over 4.6 million hospital admissions that lead to surgical care [1]. Although mortality after elective surgery is low (about 1.4 % for in-hospital mortality [2] and about 0.4 % for overall mortality [3]), surgery is an important cause of death owing to the large number of procedures. Furthermore, up to 75 % of patients experience morbidity [3], which negatively influences the quality of life.

Surgery, anaesthesia and other perioperative interventions cause trauma to tissues and physiological disturbances. Patient outcome is influenced by the type and extent of surgical insult, patient susceptibility to postoperative harm and the quality of perioperative care, although increasing age, specific co-morbidities and patient fitness are also important factors [4]. Being unfit increases the risk of death and complications after major surgery [5–7].

Enhanced recovery after surgery (ERAS) programmes, also known as ‘fast-track surgery’, are now part of standard care pathways to improve postoperative outcomes [8]. However, improving patients’ fitness prior to surgery may also lead to more positive outcomes and reduce complications [9]. ‘Prehabilitation’ is a broad term that applies to any intervention administered before surgery which aims to improve a patient’s health and fitness in order to reduce surgery-related morbidity, decrease the length of hospital stay and facilitate the patient’s return to normal. Incorporating such interventions within standard ERAS programmes may further improve outcomes for surgical patients.

## Description of the intervention

The optimal prehabilitation intervention has not been defined, but it is likely to be multicomponent, including exercise, diet, psychological and clinical components. Most prehabilitation interventions reported in the literature have focused on exercise regimens (endurance- and strength-training exercises) and have been administered in different populations awaiting elective surgery (including cardiac, cancer and orthopaedic). The studies reporting these exercise interventions are generally small and have varying results. Most have focused on patients undergoing elective orthopaedic surgery, who have an average waiting time of 18 weeks before surgery [10, 11]. In contrast, few studies have focused on cancer patients, for whom the time window to intervene is usually less than 6 weeks. The suitability of some interventions may therefore depend on the amount of time available prior to surgery.

Interventions based on exercise alone may not be sufficient to enhance functional capacity if factors such as nutrition, anxiety and perioperative care are not taken into consideration. More recent studies have included nutritional, psychological, educational and monitoring components in the prehabilitation intervention, to create a multicomponent intervention [12]. Additional components, such as smoking cessation, reducing alcohol intake and blood glucose control, may also have the potential to improve the surgical outcome and could therefore be part of multicomponent intervention.

It is unclear which components or combinations of components would form the most effective (and cost effective) prehabilitation intervention. It is also unclear whether prehabilitation interventions can be generic (i.e. successfully used in all patients undergoing elective surgery of any type) or whether they need to be tailored to patients undergoing a specific type of surgery (e.g. surgery for cancer). Patients diagnosed with potentially life-limiting conditions (such as cancer) have this emotional burden to deal with in addition to concerns about their surgery and recovery, so their care may need to have a more psychological element, for example.

## How the intervention might work

Physical fitness, nutritional status and lifestyle factors such as smoking and pre-existing co-morbidities are important prognostic factors for predicting adverse outcomes and mortality in surgical patients. Therefore, assessing baseline status and taking steps to improve these risk factors should improve outcomes for surgical patients. The mechanisms for these processes will vary depending on the type of intervention, for example, exercise improves lung function so that the patient can increase their respiratory volume to cope with increased postoperative metabolic rate, elevated body temperature and possible infections.

## Why is it important to do this review?

There is currently no consensus as to the optimal prehabilitation intervention. The term ‘prehabilitation’ has largely been used to refer to physical activity interventions, and existing systematic reviews have focused on this aspect. Currently, there are six published systematic reviews [13–18] that have investigated various physical activity regimens on clinical outcomes and health-related quality of life. Two reviews included randomised controlled trials (RCTs) only (6 to 8 studies) [13, 15], and four included both RCTs and non-RCTs (up to 21 studies) [14, 16–18]. Patient populations for these reviews were patients undergoing all adult surgery (including orthopaedic surgery) [13, 16]; elective major abdominal surgery (colorectal, liver, pancreatic, biliary) [15]; cardiac [18], respiratory or gastrointestinal surgery [14]; and surgery for cancer [17]. There is also a published protocol for a systematic review registered with the PROSPERO international prospective register of systematic reviews, also focused on physical activity interventions in all adult surgical populations [19].

A recent Cochrane systematic review assessed the effectiveness of preoperative nutrition support in patients undergoing gastrointestinal surgery [20], including RCTs assessing the effect of nutritional formulas delivered by a parenteral route, enteral route or oral supplements, most administered for 10 days preoperatively. Thirteen RCTs were included in the review. Seven evaluated immune-enhancing nutritional formulas (which reduced total postoperative complications, risk ratio (RR) 0.67, 95 % confidence interval (CI) 0.53 to 0.84), and three evaluated parenteral nutrition (also showing a reduction in postoperative complications, RR 0.64, 95 % CI 0.46 to 0.87), mainly in malnourished individuals. The remaining RCTs evaluated enteral nutrition and standard oral supplements and demonstrated no benefit. However, most of the studies included in the meta-analyses (in particular the parenteral nutrition studies) are not relevant to current practice since most protocols for the perioperative management of surgical patients do not recommend these interventions any more.

There is also a recently published Cochrane review that assessed the effectiveness of preoperative smoking interventions on smoking cessation at the time of surgery [21]. This identified seven RCTs that examined the effect of smoking-cessation interventions (including pharmacotherapy such as nicotine replacement) on postoperative complications. Two of these RCTs involved intense interventions (face-to-face or telephone counselling) and five involved brief interventions (e.g. one counselling session before surgery and additional telephone support during normal working hours if patients needed it). There was significant heterogeneity between intensive and brief interventions, so data were analysed for these subgroups separately. Results suggested a significant effect of intensive smoking-cessation intervention (RR 0.42, 95 % CI 0.27 to 0.65) but not brief intervention (RR 0.92, 95 % CI, 0.72 to 1.19) on the risk of any postoperative complication.

Other systematic reviews have assessed preoperative inspiratory muscle training in cardiac and abdominal surgery [22], preoperative education for hip and knee replacement surgery [23] and preoperative alcohol cessation [24]. Recent studies have included other components in their prehabilitation programmes. In a pilot nonrandomised study in patients undergoing elective surgery for primary colorectal cancer, Li et al. showed that a 1-month prehabilitation programme including nutritional counselling, protein supplementation and anxiety reduction techniques in addition to a moderate exercise programme increased postoperative walking capacity (at 4 and 8 weeks after surgery) and health-related quality of life and improved recovery time after surgery [12].

We intend to carry out a systematic review to identify all RCTs assessing interventions that have been used to try to improve postoperative outcomes in patients undergoing major elective surgery. This review needs to be conducted for the following reasons:

1. Most existing systematic reviews focus on specific surgical groups. It is not clear whether pre-admission interventions are generalizable across different types of surgery.
2. There is evidence that interventions other than physical activity improve postoperative outcomes in patients undergoing surgery; some of these have not been systematically identified and included in previous reviews.
3. There is a need to update the existing systematic reviews with recent RCTs.
4. There is a need to assess the methodological quality of RCTs of all interventions administered before elective surgery.

## Objectives

This systematic review protocol will be reported in line with Preferred Reporting Items for Systematic Reviews and Meta-Analyses Protocols (PRISMA-P). The objectives of this review are to (a) identify different types of intervention (e.g. physical activity, nutritional, psychosocial, clinical) that have been used prior to surgery in patients undergoing elective surgery, (b) to evaluate the benefits and harms of these interventions and (c) to compare the effectiveness of the different components.

## Methods

### Criteria for considering studies for this review

#### Types of studies

All published and unpublished RCTs will be included. Observational study designs were considered for inclusion, as they have been included in three previous systematic reviews of physical activity interventions. Observational studies were not included for the following reasons: sufficient RCTs are known to be available for different types of intervention (e.g. physical activity, nutritional, smoking cessation, multicomponent), all of which include the outcomes of interest, and observational studies are likely to be at higher risk of bias and confounding by indication, because interventions may be administered to certain subgroups of patients based on their risk of postoperative morbidity.

#### Types of participants 

Studies on adult patients (18 years and older) undergoing elective surgery (curative or palliative), not including day case surgery, will be included. We decided not to restrict the population to specific subgroups of surgery patients (e.g. cancer) because there are a limited number of RCTs available overall and we want to characterise all of these to determine whether they are transferrable or subgroup specific.

#### Types of interventions

All studies that have assessed the effectiveness of any intervention administered before elective surgery aimed at improving short-term (up to 3 months) postoperative outcomes compared with no intervention (or usual care) will be included. These could include (but are not restricted to) the following types of interventions:

- Physical activity (e.g. strength training, aerobic, specific exercises relating to the area being operated on).
- Nutritional (e.g. diet plans for weight loss or optimising nutrition for malnourished patients, oral supplementation, including macro- and micronutrients and immunonutrition). We will not include studies involving the administration of enteral and parenteral nutrition.
- Educational (e.g. advice, guidance, education, self-care strategies).
- Psychological (e.g. anxiety reducing, any cognitive or behavioural intervention or combined cognitive behavioural intervention).
- Clinical (e.g. optimising medication, diabetes/blood glucose control, treating anaemia, interventions designed to obtain a good ‘baseline’ status).
- Smoking-, alcohol- and drug-cessation/reduction interventions.
- Multicomponent (e.g. including one or more of the above).

This list is not exhaustive, and any other interventions administered preoperatively to improve postoperative outcomes identified through the literature searches will be included. We will not impose a time limit on the length of these interventions, but any intervention administered less than 24 h before surgery, or after the patient has been admitted to hospital, will be excluded. We will also exclude studies in which the interventions were continued postoperatively. Studies that compare ERAS versus no ERAS will be excluded, even if the ERAS programme includes a prehabilitation component.

### Justification for the inclusion of multiple interventions in the review

It can be argued that the proposed systematic review is ambitious given the large number of potential interventions, each of which could constitute a separate review. However, an initial scoping of the literature did not identify a large number of RCTs for the different interventions. Physical activity and nutritional and smoking-cessation interventions were most commonly identified, and there are existing systematic reviews of these interventions. Although we considered updating existing systematic reviews to prevent duplication of research effort, we decided that it would be more efficient to extract data in house for this review. Most published reviews include specific surgical populations, rather than considering major elective surgery generically. All have different inclusion and exclusion criteria, for example, some include quasi-randomised studies and in-hospital interventions, which are exclusion criteria for our review. Also, outcomes of interest are not consistently reported, and we would need to refer to the original publications for all studies included in these reviews. There may also be inconsistency in data extraction and risk of bias assessment between different reviewers. Nevertheless, we will be comparing the results of our subgroup analyses with the results from existing reviews and referencing these in our review.

### Text mining to identify additional relevant interventions

A search based on the above components may miss important interventions that have the potential to influence surgical outcome. A standard approach was used to develop the search strategy, including the reading of background literature and relevant papers, examining the search syntax of similar systematic reviews and using the clinical knowledge of relevant terms. However, because the topic is broad and multidisciplinary and may be inconsistently referred to in the literature, there is a risk that important evidence that uses different terminology will be missed. Therefore, if time and resources permit, text mining will be used alongside standard search term development methods to identify relevant search terms. Relevant full-text papers and reviews identified through our traditional search will be run through a term-extraction programme such as TerMine to identify additional key terms and compound terms, which will be added to the existing search strategies.

#### Types of outcome measures

##### Primary outcomes

- Postoperative complications: infective (e.g. chest infections, pneumonia, wound infections); non-infective (e.g. anastomotic leak, wound dehiscence, organ failure or thromboembolism)
- Length of hospital stay
- All cause perioperative mortality (30 days)

##### Secondary outcomes

- Length of stay in intensive care unit (ICU) and/or high dependency unit (HDU)
- Perioperative morbidity (acute coronary event, stroke)
- Hospital readmission
- Postoperative pain
- Health-related quality of life (HRQoL)
- Outcomes specific to intervention: physical activity (e.g. forced expiratory volume in 1 s (FEV1); cardiopulmonary exercise testing (CPET); muscle function tests such as 6-min walk tests, muscle strength and motor function, inspiratory muscle function), nutritional (e.g. body weight, body composition, body mass index, biochemical markers such as serum albumin), psychological (e.g. anxiety, distress, depression, fatigue), clinical (e.g. mean blood glucose during intervention), alcohol/drug cessation (reported abstinence/reduction, biochemical validation)
- Any reported adverse effects
- Resource use

### Search methods for identification of studies

The following electronic databases were used to identify relevant trials. Searches were not restricted by language or publication status. Searches were conducted on 9 March 2015.

1. MEDLINE and PreMEDLINE (OvidSP) (1950 to date)
2. EMBASE Classic + EMBASE (OvidSP) (1974 to date)
3. CENTRAL, DARE, HTA and NHS EED (The Cochrane Library, latest issue)
4. CINAHL (1981 to date)
5. PsycINFO (1806 to date)
6. ISI Web of Science: Science Citation Index Expanded (SCIEXPANDED) (1900 to date)
7. ISI Web of Science: Conference Proceedings Citation Index-Science (CPCI-S) (1990 to date).

We considered whether to search complementary and alternative medicine (CAM) databases to identify CAM interventions (e.g. homoeopathy, acupuncture, osteopathy, yoga, herbalism) that have been used in patients before surgery to improve outcomes but decided not to do so for the following reasons: (a) the methodological and reporting quality of CAM trials is generally poor [25, 26], so it is unlikely that conclusions could be drawn from these studies and that any CAM interventions could be incorporated into a prehabilitation intervention; (b) there is a growing body of evidence that questions the effectiveness of CAM therapies and their underlying theories [27]; and (c) there are limited resources and time available to complete this review.

### Searching other resources

All the reference lists of all included studies and published systematic reviews will be hand searched. Databases of ongoing trials will also be searched: Current Controlled Trials (www.controlled-trials.com with links to other databases of ongoing trials) and the WHO International Clinical Trials Registry Platform (www.who.int/ictrp/en/). Grey literature databases (e.g. OpenGrey); the Google search engine and a combination of key text words to identify studies published in non-indexed journals, theses and dissertations; and published POA protocols will also be used. Experts in the field and trial authors will be contacted for further information or unpublished data. The search strategy for MEDLINE is shown in the Appendix. This was adapted as appropriate for searching the other databases.

### Data collection and analysis

#### Selection of studies

Two review authors will independently screen titles and abstracts to determine eligibility. Full-text papers will be obtained for all studies deemed eligible or studies that do not provide sufficient information to be excluded at the screening stage. All full-text papers will be assessed for eligibility by two review authors independently; studies not meeting the inclusion criteria will be excluded, and the reasons for exclusion will be recorded. Disagreements will be resolved by discussion and consensus with a third review author. The study selection process will be presented in a PRISMA flow diagram.

#### Data extraction and management

The following information will be extracted from each study:

1. Publication details (authors, title, date of publication, country of origin, funding source, corresponding author contact details).
2. Study characteristics (setting, study design, method of randomisation (sequence generation, concealment of allocation), blinding of outcome assessors, number of patients randomised to each group).
3. Participant characteristics (demographics, inclusion and exclusion criteria, clinical characteristics (e.g. type and extent of disease, presence of co-morbidities, proportion of malnourished patients (defined by body mass index <20 kg/m^2^) and proportion of overweight or obese patients (defined by body mass index >25 or >30 kg/m^2^, respectively), subjective global assessment or nutrition risk derived from a validated tool, functional capacity parameters)).
4. Surgery characteristics (e.g. type of surgery, perioperative management (e.g. ERAS or traditional)).
5. Intervention and comparator characteristics. These will be prespecified for each type of intervention. We will also extract information from all included studies on the following factors: mode of delivery (verbal, written, computer, phone app), place of delivery (hospital, home, other), who delivered the intervention (doctor, nurse, other health professional), training for individuals delivering the intervention (yes/no), duration of the intervention, number of sessions, format (individual or group), level of commitment required from the patient (high/medium/low) and acceptability of the intervention to the patient. We will extract information in compliance with the intervention.
6. Outcomes (as detailed in the previous section). We will record the number of participants assessed for each outcome, the mean values and standard deviations (if available) or medians and interquartile ranges for continuous data, the number of events in each group for categorical data, and any reported summary statistics (e.g. effect estimates, confidence intervals (CIs), standard errors (SEs), ranges). We will contact the trial authors for information if any of the above data items are missing.

#### Dealing with duplicate publications

Where multiple papers have reported the same study but different outcomes, all will be used to extract the relevant outcome data.

#### Assessment of risk of bias in included studies

The risk of bias for each included study will be assessed independently by at least two of the three review authors. We will use The Cochrane Collaboration’s tool [28] for assessing the risk of bias and rate the quality of each trial (low risk, unclear and high risk) in the following areas: generation of allocation sequence (selection bias); allocation concealment (selection bias); blinding of participants, personnel and outcome assessors (performance bias and detection bias); incomplete outcome data (attrition bias); selective reporting (reporting bias); and other sources (e.g. presentation data bias, sampling bias, sponsorship bias).

Blinding of participants and health care professionals in trials involving physical activity interventions, dietary changes (unless it involves oral supplementation), educational and psychological interventions and smoking/alcohol/drug-cessation/reduction interventions is likely to be difficult, and complete blinding may not be possible. To account for outcome-specific variation in performance bias, detection bias and selective outcome reporting bias, the risk of bias will be assessed separately for the following prespecified outcome domains: perioperative mortality, hospital readmission, postoperative complications and morbidity, length of stay (ITU/HDU/hospital), patient-reported outcomes (postoperative pain, HRQoL, psychological outcomes) and clinical measurements (e.g. cardiopulmonary testing parameters, muscle function tests, BMI, biomarkers). Different criteria will be used to assess the risk of bias for each domain, so for example, the lack of blinding of outcome assessors is less likely to influence outcomes such as mortality and hospital readmission, so these outcomes will be judged to be at a low risk of detection bias, whereas lack of any attempt to blind participants is likely to influence patient-reported outcomes, so these outcomes will be judged to be at a high risk of detection bias. Similarly, clinical measurement outcomes will be judged to be at a low risk of detection bias if the outcome assessors are blinded, regardless of whether or not patients and personnel are blinded. We will assess the strength of the overall body of evidence for each outcome domain using the Grading of Recommendations Assessment, Development and Evaluation (GRADE) methodology [29].

#### Assessment of adverse events in included studies

An assessment of the effectiveness of an intervention requires evidence of both benefits and harms. Although RCTs may be poor at identifying and reporting the harms of an intervention [30, 31], any additional information about adverse events that may be related to the intervention and assess the risk of bias as for the other outcomes will be extracted. In addition, patient risk factors and the length of follow-up when reviewing adverse events will also be considered, as will the risk of reporting bias for adverse events.

#### Measures of treatment of effect

For dichotomous outcomes (mortality, morbidity, hospital readmission, adverse events and most postoperative complications), we will calculate pooled risk ratios (RRs) and 95 % confidence intervals (CIs). For continuous outcomes (most patient-reported outcomes, physiological and clinical parameters), we will calculate pooled mean differences and 95 % CIs when results are reported on the same scale (or can be converted to the same scale) or standardised mean differences and 95 % CIs if results are reported on different scales. The length of ITU/HDU/hospital stay, although strictly speaking time-to-event data, are often reported as continuous data despite the fact that such data are unlikely to be normally distributed. However, if authors report medians and interquartile ranges (allowing for censoring), we will calculate hazard ratios (HRs) and 95 % CIs. If no appropriate data are available, then the length of stay will be reported narratively.

#### Unit of analysis issues

We will take into account multiple observations for the same outcome and the level at which randomisation occurred, although we are not aware of any cluster randomised trials.

#### Dealing with missing data

If the data required are not available in the publication, we will first attempt to back-calculate from the data presented (e.g. numerator or denominator from percentages, standard deviation from standard errors or 95 % CIs). If this is not possible, we will attempt to contact the study authors. We are aware that studies assessing lifestyle interventions may have issues with compliance; therefore, we will carefully report reasons for missing data (e.g. dropouts, losses to follow-up and withdrawals).

#### Assessment of heterogeneity

We will assess clinical heterogeneity across studies by examining the variability in the details of participants, baseline data, interventions and outcomes to determine whether studies are similar. Statistical heterogeneity will be quantified using the *I*^2^ statistic; we will consider the statistical heterogeneity to be high if *I*^2^ > 50 % [28].

We will attempt to explain any observed clinical or statistical heterogeneity in the results of the review.

#### Reporting biases

Funnel plots will be used to assess publication bias when ten or more studies are included in a meta-analysis.

#### Data synthesis

We will attempt to combine the results for trials that use similar interventions (e.g. physical activity, nutritional, educational, psychological, clinical) and report similar outcomes. However, even with similar interventions, there is likely to be substantial heterogeneity in the types of participants, the intervention and its delivery. We will therefore be using random-effect meta-analysis models for our primary analysis to pool data across trials, although fixed-effect meta-analysis models will also be explored. The findings from the included studies will be summarised in narrative form if we do not find trials that are sufficiently similar to justify a meta-analysis.

#### Subgroup analyses and investigation of heterogeneity

If data from sufficient trials are available, we will attempt to perform subgroup analyses for the following subgroups:

1. Type of surgery (e.g. orthopaedic, cardiac, cancer)
2. Type of intervention (e.g. intensive or brief)
3. Intervention conducted pre- or post-ERAS
4. High- vs low-risk surgical patients

#### Sensitivity analyses

We will conduct sensitivity analyses to include only the trials classified as having good allocation concealment. Sensitivity analysis excluding trials with more than 20 % dropout rate will be performed to assess the impact of the missing data on our results and conclusions. The impact of removing any study that has a large effect size from the meta-analyses will also be assessed.

## Appendix

### Search strategy

#### Database: MEDLINE In-process - Current week, MEDLINE 1950 to present

Search Strategy:

1 (pre-hab$ or prehab$).ti,ab. (119)

2 ((presurg$ or preoperativ$ or pre-surg$ or pre-operativ$) adj3 (conditioning or optimis$ or optimiz$ or rehab$ or re-hab$ or care)).ti,ab. (1790)

3 (pre adj2 (postsurg$ or postoperativ$) adj3 (conditioning or optimis$ or optimiz$ or rehab$ or re-hab$ or care)).ti,ab. (406)

4 ((before or prior to) adj3 (CABG or surgery or surgical or procedure$ or arthroplast$ or hip replacement or knee replacement or joint replacement or total hip or total knee or total joint$ or operation) adj12 (conditioning or optimis$ or optimiz$ or rehab$ or re-hab$ or care)).ti,ab. (1137)

5 Preoperative Care/mt, rh [Methods, Rehabilitation] (9939)

6 (preoperative care/ or preoperative period/) and (conditioning or optimis$ or optimiz$ or rehab$ or re-hab$).ti,ab. (1435)

7 Postoperative Complications/pc and ((presurg$ or preoperativ$ or pre-surg$ or pre-operativ$) adj3 (assess$ or intervention$)).ti,ab. (349)

8 or/1-7 (14185)

9 preoperative care/ or preoperative period/ (54529)

10 (presurg$ or preoperativ$ or pre-surg$ or pre-operativ$).ti,ab. (224877)

11 (pre adj2 (postsurg$ or postoperativ$)).ti,ab. (12194)

12 ((before or prior to) adj3 (CABG or surgery or surgical or procedure$ or hip replacement or knee replacement or joint replacement or total hip or total knee or total joint$ or arthroplast$ or operation)).ti,ab. (83657)

13 or/9-12 (316706)

14 exp Exercise/ or exp Exercise Therapy/ (146992)

15 physical therapy modalities/ (28521)

16 physical fitness/ (22302)

17 (exercis$ or aerobic$ or swim$ or hydrotherapy or preconditioning or pre-conditioning or physical fitness or physical activit$ or physiotherap$ or physical therap$).ti,ab. (365238)

18 ((muscle or endurance or resistance or weight or strength) adj2 training).ti,ab. (13555)

19 ((function or functional capacity) adj2 (enhanc$ or improv$ or maximis$)).ti,ab. (39597)

20 Nutrition Therapy/ or exp diet therapy/ or exp diet/ or eating/ or nutritional physiological phenomena/ or elder nutritional physiological phenomena/ or nutritional requirements/ or nutritional status/ (288993)

21 exp Dietary Supplements/ or exp Food, Fortified/ (50721)

22 exp Malnutrition/dh, pc, rh, th [Diet Therapy, Prevention & Control, Rehabilitation, Therapy] (12552)

23 (diet$ or nutrition$ or malnutrition or underweight or low BMI or undernourish$ or undernutrition or malnourish$ or immunonutrition or macronutrient$ or micronutrient$ or immuno-nutrition or macro-nutrient$ or micro-nutrient$).ti,ab. (568387)

24 ((oral or food or multinutrient$ or multi-nutrient$ or multivitamin$ or iron or protein or folate or vitamin$) adj3 supplement$).ti,ab. (33003)

25 ((iron or protein or folate or vitamin$) adj3 (deficient or deficiency)).ti,ab. (47403)

26 ((fortif$ or enrich$) adj3 (food$ or feed$)).ti,ab. (3276)

27 (weight adj2 (loss or lose$ or lost or losing) adj2 (program$ or plan$)).ti,ab. (1818)

28 (fortisip or complan).ti,ab. (17)

29 Adaptation, Psychological/ (76095)

30 Cognitive Therapy/ or Psychotherapy/ or exp Mind-body therapies/ or behavior therapy/ or mindfulness/ (116265)

31 ((counselling or counseling) adj2 (session$ or therap$ or intervention$)).ti,ab. (3428)

32 mindfulness.ti,ab. (2121)

33 (CBT or ((cognitive or talking or mental health or behavio?ral) adj3 (intervention$ or therap$))).ti,ab. (27170)

34 ((education$ or psychoeducational or psychotherapeutic or psychological or psychosocial or behavio?ral or cognitive) adj3 (intervention$ or program$)).ti,ab. (67734)

35 ((anxiety or stress or fear) adj2 (manag$ or strateg$ or therap$ or reduc$)).ti,ab. (24159)

36 ((selfcare or self-care or self-help or coping) adj2 (mechanism$ or strateg$ or behavio?r$)).ti,ab. (13440)

37 Smoking Cessation/ (20803)

38 ((smoking or drug$) adj2 (cessation or stop$ or quit$ or giving up or give up)).ti,ab. (24592)

39 (nicotine replacement therapy or NRT).ti,ab. (2339)

40 (alcohol adj2 reduc$).ti,ab. (4053)

41 Blood Glucose Self-Monitoring/ (4398)

42 ((blood sugar or blood glucose or diabetes) adj2 (level or levels or control$ or monitor$)).ti,ab. (32523)

43 Anemia/dt, pc, th [Drug Therapy, Prevention & Control, Therapy] (10586)

44 ((an?emia or an?emic) adj3 (iron or prevent$ or treat$ or control$)).ti,ab. (16266)

45 ((blood pressure or BP or hypertens$) adj2 (control$ or manag$ or medication$)).ti,ab. (28359)

46 ((COPD or angina) adj2 (control$ or manag$ or medication$)).ti,ab. (2158)

47 Geriatric Assessment/ (19045)

48 ((geriatric or baseline status) adj2 assessment$).ti,ab. (2248)

49 ((optimiz$ or optimis$) adj2 medication$).ti,ab. (251)

50 (pre-existing adj2 (comorbidit$ or co-morbidit$ or chronic illness$ or chronic disease$ or chronic condition$)).ti,ab. (233)

51 or/14-50 (1543244)

52 8 or (13 and 51) (32833)

53 letter/ (867912)

54 editorial/ (371347)

55 news/ (167087)

56 exp historical article/ (328799)

57 Anecdotes as topic/ (4603)

58 comment/ (615244)

59 case report/ (1715831)

60 (letter or comment$).ti. (101137)

61 or/53-60 (3438065)

62 randomized controlled trial/ or Randomized Controlled Trials as Topic/ or random$.ti,ab. (896854)

63 61 not 62 (3406560)

64 animals/ not humans/ (3906384)

65 exp Animals, Laboratory/ (735772)

66 exp Animal Experimentation/ (6519)

67 exp Models, Animal/ (428521)

68 exp rodentia/ (2704363)

69 (rat or rats or mouse or mice or animal or animals).ti. (1219737)

70 or/63-69 (7953941)

71 52 not 70 (28469)

72 (exp child/ or exp infant/) not adult/ (1398640)

73 ((child$ or infant$ or newborn$ or neonat$) not adult$).ti. (826483)

74 72 or 73 (1610061)

75 71 not 74 (26156)

76 meta-analysis/ (53771)

77 meta-analysis as topic/ (14025)

78 (meta analy$ or metaanaly$ or metanaly$ or meta regression).ti,ab. (73844)

79 ((systematic$ or evidence$) adj2 (review$ or overview$)).ti,ab. or review.ti. (315502)

80 (reference list$ or bibliograph$ or hand search$ or manual search$ or relevant journals).ab. (27398)

81 (search strategy or search criteria or systematic search or study selection or data extraction).ab. (29241)

82 (search$ adj4 literature).ab. (31134)

83 (medline or pubmed or cochrane or embase or psychlit or psyclit or psychinfo or cinahl or science citation index or bids or cancerlit).ab. (96758)

84 cochrane.jw. (11169)

85 ((multiple treatment$ or indirect or mixed) adj2 comparison).ti,ab. (1006)

86 or/76-85 (435094)

87 randomized controlled trial.pt. or randomized controlled trial/ or Randomized Controlled Trials as Topic/ (477876)

88 controlled clinical trial.pt. (88869)

89 ((doubl$ or singl$ or trebl$ or tripl$) adj blind$).ti,ab. (130373)

90 random$.ti,ab. (749676)

91 clinical trials as topic.sh. (171370)

92 trial.ti. (134197)

93 (controlled adj clinical trial).ti,ab. (9292)

94 or/87-93 (1143831)

95 75 and (86 or 94) (4843)

## Abbreviations

CI: confidence interval
ERAS: enhanced recovery after surgery
RCT: randomised controlled trial
RR: risk ratio
